# *BrAN* contributes to leafy head formation by regulating leaf width in Chinese cabbage (*Brassica rapa* L. ssp. *pekinensis*)

**DOI:** 10.1093/hr/uhac167

**Published:** 2022-07-27

**Authors:** Yue Xin, Chong Tan, Che Wang, Yanji Wu, Shengnan Huang, Yue Gao, Lu Wang, Nan Wang, Zhiyong Liu, Hui Feng

**Affiliations:** Liaoning Key Laboratory of Genetics and Breeding for Cruciferous Vegetable Crops, College of Horticulture, Shenyang Agricultural University, Shenyang 110866, China; Liaoning Key Laboratory of Genetics and Breeding for Cruciferous Vegetable Crops, College of Horticulture, Shenyang Agricultural University, Shenyang 110866, China; College of Bioscience and Biotechnology, Shenyang Agricultural University, Shenyang 110866, China; Liaoning Key Laboratory of Genetics and Breeding for Cruciferous Vegetable Crops, College of Horticulture, Shenyang Agricultural University, Shenyang 110866, China; Liaoning Key Laboratory of Genetics and Breeding for Cruciferous Vegetable Crops, College of Horticulture, Shenyang Agricultural University, Shenyang 110866, China; Liaoning Key Laboratory of Genetics and Breeding for Cruciferous Vegetable Crops, College of Horticulture, Shenyang Agricultural University, Shenyang 110866, China; College of Bioscience and Biotechnology, Shenyang Agricultural University, Shenyang 110866, China; Liaoning Key Laboratory of Genetics and Breeding for Cruciferous Vegetable Crops, College of Horticulture, Shenyang Agricultural University, Shenyang 110866, China; Liaoning Key Laboratory of Genetics and Breeding for Cruciferous Vegetable Crops, College of Horticulture, Shenyang Agricultural University, Shenyang 110866, China; Liaoning Key Laboratory of Genetics and Breeding for Cruciferous Vegetable Crops, College of Horticulture, Shenyang Agricultural University, Shenyang 110866, China; Liaoning Key Laboratory of Genetics and Breeding for Cruciferous Vegetable Crops, College of Horticulture, Shenyang Agricultural University, Shenyang 110866, China

## Abstract

Leafy head is an important agronomic trait that determines the yield and quality of Chinese cabbage. The molecular mechanism underlying heading in Chinese cabbage has been the focus of research, and wide leaves are a prerequisite for leafy head formation. In our study, two allelic leafy heading-deficient mutants (*lhd1* and *lhd2*) with narrow leaf phenotypes were screened in an ethyl methanesulfonate mutagenized population from a heading Chinese cabbage double haploid line ‘FT’. Genetic analysis revealed that the mutant trait was controlled by a recessive nuclear gene, which was found to be *BraA10g000480.3C* by MutMap and Kompetitive allele-specific PCR analyses. As *BraA10g000480.3C* was the ortholog of *ANGUSTIFOLIA* in *Arabidopsis*, which has been found to regulate leaf width by controlling cortical microtubule arrangement and pavement cell shape, we named it *BrAN*. *BrAN* in mutant *lhd1* carried an SNP (G to A) on intron 2 that co-segregated with the mutant phenotype, and disrupted the exon-intron splice junction generating intron retention and a putative truncated protein. *BrAN* in mutant *lhd2* carried an SNP (G to A) on exon 4 leading to a premature stop codon. The ectopic overexpression of *BrAN* restored normal leaf phenotype due to abnormal cortical microtubule arrangement and pavement cell shape in the *Arabidopsis an-t1* mutant. However, transformation of *Bran* did not rescue the *an-t1* phenotype. These results indicate that *BrAN* contributes to leafy head formation of Chinese cabbage.

## Introduction

Chinese cabbage (*Brassica rapa* L. ssp. *pekinensis*) is a leafy vegetable crop widely cultivated in Eastern Asia, and the leafy head determines its commercial value. Vegetative growth for Chinese cabbage is divided into four stages, including the seedling, rosette, folding, and heading stages. At the seedling stage, the leaves grow flat. At the rosette stage, leaves are large and round, providing the basis for vegetative growth through photosynthesis [1]. After the rosette stage, leaves grow upward and inward and form a leafy head. The molecular mechanism responsible for heading has been the focus of research on Chinese cabbage.

Leafy head formation in Chinese cabbage is a complex biological process. Many factors are involved in this process, such as temperature, light intensity, auxin distribution, and the ratio of carbon to nitrogen [2]. Differences in auxin concentrations and its distribution in the adaxial and abaxial side of leaves make them curl inward, which promotes leafy head formation [1, 3]. Leafy head development may correlate to the regulation of transcription factors, protein kinases, and calcium [4]. Some adaxial-abaxial patterning genes have been found to be involved in leaf incurvature [5]. Leafy heads consist of leaves arranged tightly around each other. The key transition leaves have been identified for heading by series-spatial transcriptome profiling of leafy heads [6]. In addition, ample evidence has shown that leafy head traits are closely related to leaf traits in Chinese cabbage, such as leaf length [7], width [8], size [9–11], number [12], curvature [9, 13, 14], and angle to the ground [10, 15]. Sun et al. [8] have shown that width of rosette leaves at different developmental stages is correlated with the size and degree of leafy heads, indicative of the crucial effects of leaf width.

Several parameters at the cellular level, including cell number, size, shape, and positioning can define leaf morphology [16]. It is important to explore how these changes occur at the cellular level and why the parameters change. Cortical microtubule arrangement determines directional cell expansion and plant morphogenesis by controlling cellulose deposition [17, 18]. Cortical microtubules mediate cellulose deposition along the main predicted stress orientations, especially along the adaxial-abaxial axis in the internal cell walls to determine leaf shape [19]. As a cytoskeleton component, tubulin is important in maintaining cell morphology. In addition, microtubule-associated proteins and microtubule-arrangement-associated proteins play a crucial role in cell morphology. OsIQ67-DOMAIN14, a microtubule-associated protein, regulates cell shape and yield in rice [20]. BRASSINOSTEROID INSENSITIVE2, a negative regulator of brassinosteroid responses in plants, regulates pavement cell growth and leaf development by stabilizing microtubules [21].

**Figure 1:**
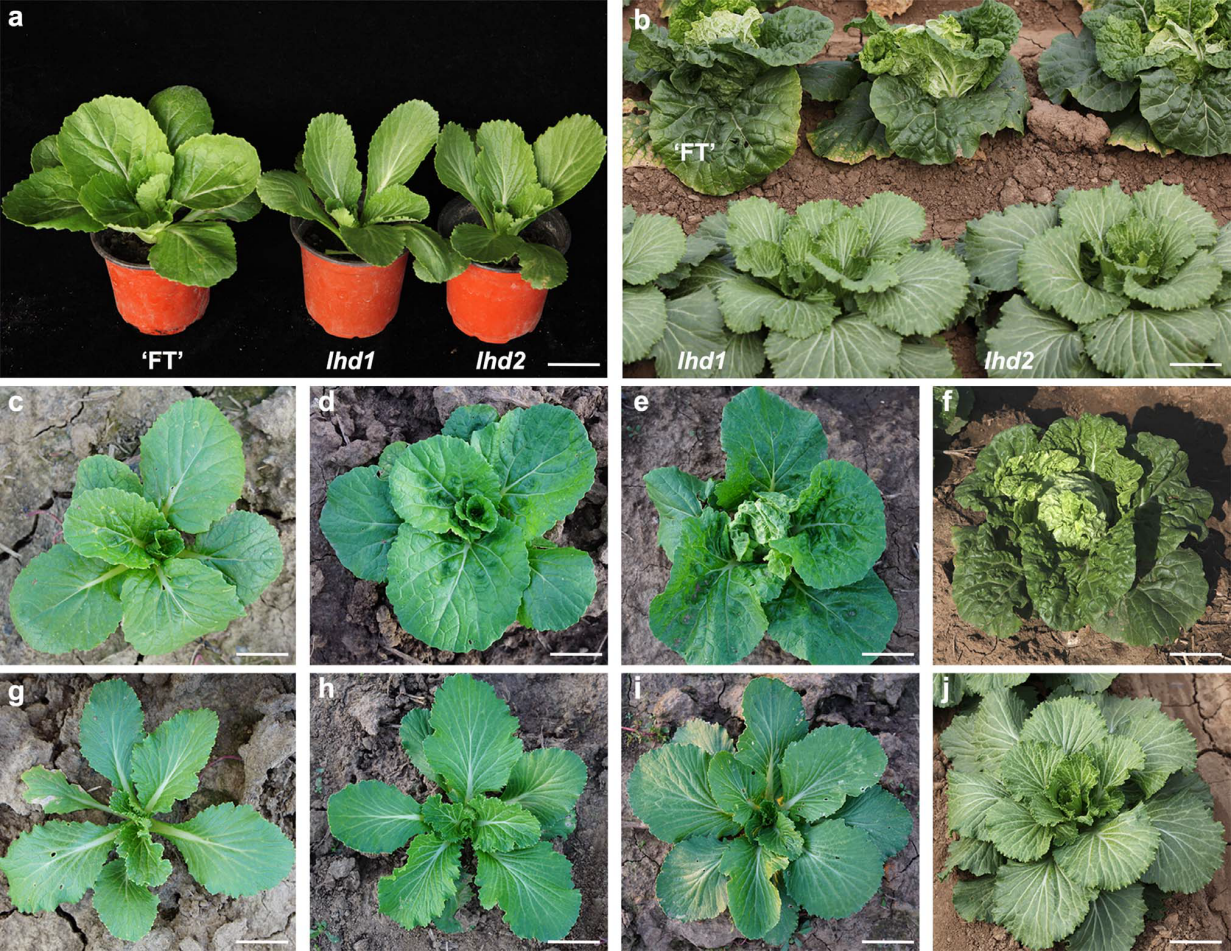
Phenotypic characterisation. (**a**, **b**) Morphological characteristics of *lhd1*, *lhd2*, and their wild-type ‘FT’. Scale bar = 2 cm (**a**) and scale bar = 10 cm (**b**). (**c**–**f**) Four stages of leafy head formation in wild-type ‘FT’. Seedling stage (**c**) and rosette stage (**d**). Scale bar = 2 cm. Folding stage (**e**) and heading stage (**f**). Scale bar = 10 cm. (**g**–**j**) Four stages in mutant compared with wild-type ‘FT’. Seedling stage (**g**) and rosette stage (**h**). Scale bar = 2 cm. Folding stage (**i**) and heading stage (**j**). Scale bar = 10 cm.

*ANGUSTIFOLIA* in *Arabidopsis* (*AtAN*) encodes a plant homologue of C-terminal binding protein (CtBP) in animals, which is involved in several biochemical pathways. The characterization of *angustifolia* (*an*) mutation includes narrow leaves, fewer trichome branches, and twisted seed pods, petals, and roots [22, 23]. There have been numerous recent reports on *AtAN* involvement in regulating epidermal cell differentiation and shape [24], microtubule arrangement [25, 26], conical cell shape [27], stress granule assembly [28], response to abiotic and biotic stressors [29], nuclear migration [30], and regulation of pathogenic response [31]. The abnormal cell shape of narrow leaves in *an* has been previously associated with the abnormal arrangement of cortical microtubules, which changes the polarity of cell growth.

Several leafy heading-deficient mutants with narrow leaves were screened from an ethyl methanesulfonate mutagenized population created in our study [32]. Two of them (*lhd1* and *lhd2*) were shown to be allelic via an allelism test and were selected to explore the molecular mechanism underlying leafy head formation. *BrA10g000480.3C* was identified as a candidate gene for the leafy heading-deficient mutation via MutMap, Kompetitive allele-specific PCR (KASP), and comparative sequencing analysis. The cortical microtubule arrangement and pavement cell shape variations, which led to narrow leaves in the *Arabidopsis an-t1* mutant, were restored by *BrAN* overexpression*.* Our findings proved that *BrAN* mutation in Chinese cabbage caused the leafy head deficiency, and suggested that leaf width is critical for leafy head formation.

## Results

### Phenotypic characterisation and allelic test of *lhd1* and *lhd2*

The mutant leaves grew flat and outward and were narrower and thicker than those of the wild-type ‘FT’ from the seedling stage to the heading stage. An allelism test between *lhd1* F_1_ and *lhd2* F_1_ indicated that 77 and 23 plants exhibited wild-type and mutant phenotypes, respectively. The 3:1 segregation of F_2_ population (*P* = 0.12 in χ2 test) suggested that *lhd1* and *lhd2* mutation were controlled by one allelic gene (Fig. 1a, b).

The true leaves of the mutant exhibited smooth surfaces without wrinkles and straighter primary veins with less secondary branching than ‘FT’. The mutant and ‘FT’ plant architecture was similar until the rosette stage (Fig. 1c, d; g, h). At the folding stage, ‘FT’ leaves folded upward and curled inward to form a leafy head (Fig. 1e). However, more rosette leaves, which showed no tendency to curl, were observed in the mutant (Fig. 1i). At the heading stage, leaves spreading outwards instead of a leafy head were observed in the mutant (Fig. 1f, j). Additionally, the cotyledons and petals in the mutant were narrower than those in ‘FT’ and the number of seeds per pod in the mutant was less than in ‘FT’ (Supplementary Figure S1).

**Figure 2:**
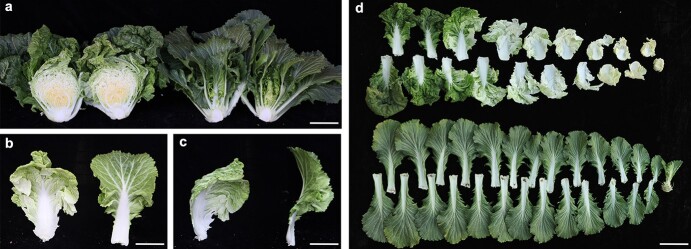
Phenotype of the mutant compared to wild-type ‘FT’ at the heading stage. (**a**) Cross-sections of ‘FT’ (left) and the mutant (right). Scale bar = 10 cm. (**b**) Observation of heading leaves of ‘FT’ (left) and the mutant (right) from the front. (**c**) Observation of heading leaves of ‘FT’ (left) and the mutant (right) from the side. Scale bar = 10 cm. (**d**) True leaves of ‘FT’ (top) and the mutant (bottom) at the heading stage. Scale bar = 10 cm.

At the mature stage (heading stage of Chinese cabbage), leaves of ‘FT’ arranged tightly around each other to form a leafy head, whereas leaves of the mutant did not overlap or form a leafy head (Fig. 2a). The heading leaf in ‘FT’ was curved inward, whereas it was curved outward in the mutant (Fig. 2b, c). Furthermore, there were more leaves observed in the mutant, and all leaves of the mutant were as dark green as the outer ones because they were exposed to light during all stages (Fig. 2d).

**Figure 3:**
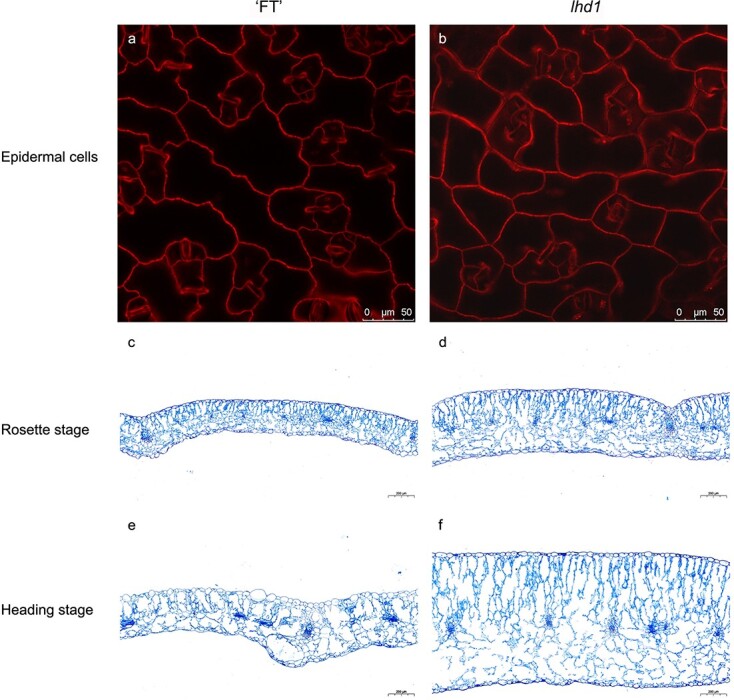
Cell phenotypes of wild-type ‘FT’ and *lhd1.* (**a**, **b**) Leaf abaxial epidermal cells of ‘FT’ (**a**) and *lhd1* (**b**) in a seedling leaf. Scale bar = 50 μm. (**c**, **d**) Leaf transverse sections of ‘FT’ (**c**) and *lhd1* (**d**) in a rosette leaf. Scale bar = 200 μm. (**e**, **f**) Leaf transverse sections of ‘FT’ (**e**) and *lhd1* (**f**) in a heading leaf. Scale bar = 200 μm.

To explore whether leaf morphological differences were related to cell morphology, we first measured the leaf length and width at the four stages and calculated the leaf index of ‘FT’ *lhd1,* and *lhd2* leaves. The leaf length of *lhd1* and *lhd2* was similar to that of ‘FT’ at all stages, while the leaf width of *lhd1* and *lhd2* was significantly less than that of ‘FT’, and the leaf index was significantly higher than that of ‘FT’ (Table 1; Supplementary Table S1). Subsequently, the epidermal cells at the seedling stage were observed. *lhd1* and *lhd2* leaves exhibited simpler adaxial epidermal cells than those of ‘FT’ (Fig. 3a, b; Supplementary Figure S2a, b). Moreover, the cell area and number in ‘FT’, *lhd1,* and *lhd2* were measured at the seedling stage. The difference in epidermal cell number and size between ‘FT’ and leafy heading-deficient mutant seedling leaves was significant (*P* < 0.05) (Supplementary Table S2).

**Table 1:** Parameters of leaves in the ‘FT’ and *lhd1*.

Plant	Length (cm)	Width (cm)	Length-to-width ratio
Seedling stage			
‘FT’	7.07 ± 0.43	4.00 ± 0.35	1.78 ± 0.13
*lhd1*	7.02 ± 0.32	2.41 ± 0.24^**^	2.93 ± 0.26^**^
Rosette stage			
‘FT’	12.68 ± 0.21	8.83 ± 0.15	1.44 ± 0.02
*lhd1*	12.71 ± 0.17	6.95 ± 0.13^**^	1.94 ± 0.02^**^
Folding stage			
‘FT’	19.00 ± 0.97	18.96 ± 1.94	1.01 ± 0.08
*lhd1*	18.84 ± 0.96	14.58 ± 0.80^**^	1.30 ± 0.06^**^
Heading stage			
‘FT’	25.00 ± 0.68	22.34 ± 0.67	1.12 ± 0.04
*lhd1*	24.81 ± 0.67	17.24 ± 0.56^**^	1.44 ± 0.05^**^

The parameters length, width, and length-to-width ratio were measured at four different stages. Values are the means ± SD (*n* = 20 seedlings, ^**^P < 0.01). Statistically significant differences were calculated based on Student’s *t*-tests

To further characterize the leaf morphology in the leaf-thickness direction, longitudinal sections of ‘FT’ and leafy heading-deficient mutant leaves were observed at the rosette and heading stages. In rosette leaves of ‘FT’, *lhd1,* and *lhd2*, a palisade parenchyma layer and a spongy parenchyma layer were observed on the adaxial and abaxial side respectively (Fig. 3c, d; Supplementary Figure S2c, d). *lhd1* and *lhd2* leaves exhibited larger cells than those of ‘FT’. At the heading stage, *lhd1* and *lhd2* epidermal cells were packed flat while those in ‘FT’ were unevenly arranged because the leaves were bent to form a leafy head (Fig. 3e, f; Supplementary Figure S2e, f). *lhd1* and *lhd2* leaves were thicker than those of ‘FT’. There was no palisade parenchyma layer in the ‘FT’ curling leaves, and the spongy parenchyma cells were packed on both the adaxial and abaxial sides, suggesting that the leaf adaxial–abaxial patterning had changed; however, the palisade and spongy parenchyma layers could still be clearly distinguished in *lhd1* and *lhd2* flatting leaves.

### Genetic characterization

The segregating populations of *lhd1* were phenotyped for leafy head. The segregation ratios are presented in Table 2. All F_1_ plants showed the wild-type phenotype. Among ‘FT’ × *lhd1* F_2_ plants, 321 and 89 showed the wild-type and mutant phenotypes, respectively. These results are consistent with a 3:1 segregation ratio (χ^2^ = 2.198). The phenotype of 25 plants from F_1_ × ‘FT’ was similar to that of ‘FT’. Among the F_1_ × *lhd1* plants observed, 18 and 20 showed wild-type and mutant phenotypes, respectively, consistent with a 1:1 segregation ratio (χ^2^ = 0.026). Phenotypic analysis indicated that the *lhd1* leafy heading-deficient phenotype was controlled by a single recessive nuclear gene. Genetic analysis of *lhd2* showed that a single recessive nuclear gene was responsible for the *lhd2* leafy heading-deficient mutation (Supplementary Table S3).

**Table 2:** Genetic analysis of leafy heading-deficient phenotype in mutant *lhd1*.

Generations	Total	‘FT’	*lhd1*	Segregation ratio	χ2
P_1_(‘FT’)	50	50	0		
P_2_(*lhd1*)	50	0	50		
F_1_(P_1_ × P_2_)	50	50	0		
F_1_(P_2_ × P_1_)	30	30	0		
BC_1_(F_1_ × ‘FT’)	40	40	0		
BC_1_(F_1_ × *lhd1*)	38	18	20	0.900:1	0.026
F_2_	410	321	89	3.607:1	2.198

**Figure 4:**
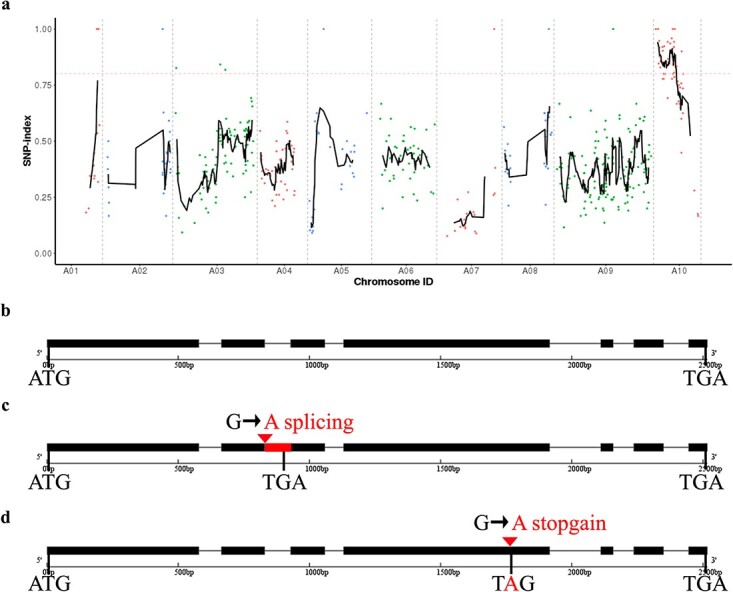
MutMap SNP index plot and *BrAN* gene structure. (**a**) SNP index plot of 10 chromosomes produced using MutMap analysis. X-axis represents positions of 10 chromosomes; Y-axis represents SNP index. Dotted pink line represents the index threshold (0.80). (**b**–**d**) Gene structure of *BrAN* in ‘FT’ (**b**), *Bran* in *lhd1* (**c**), and *lhd2* (**d**). Black boxes and lines represent exons and introns, respectively. Red box represents the second intron retention. Red triangles show sites where the causal mutation occurred in *lhd1* and *lhd2*, respectively.

### Candidate gene identification for *lhd1*

A modified MutMap approach was used for the candidate gene identification. We obtained 14 133 Mb (98.28%), 13 412 Mb (99.65%), and 19 407 Mb (99.64%) clean reads by resequencing of ‘FT’, *lhd1,* and N-pool, respectively. Subsequently, 98.00%, 99.02%, and 98.85% of them were aligned to the V3.0 reference genome, and 2 220 286 SNPs were detected. The following SNPs were removed by filtration: (i) those supporting <3 or >100 reads from ‘FT’ and *lhd1*, and <5 or >150 reads from the mutant pool; (ii) genotyping quality <20; (iii) heterozygous in ‘FT’; (iv) heterogeneous and both alleles inconsistent with ‘FT’ in the mutant pool; (v) completely consistent between ‘FT’ and *lhd1* pool; and (vi) not C → T or G → A. Homozygous SNPs with different typing in ‘FT’ and *lhd1* were conserved. Finally, 540 SNPs were obtained, and the MutMap SNP index was calculated and distributed on the chromosomes using the slide window method (Fig. 4a). We located a candidate region of 9.68 Mb (228 525–9 905 099) on chromosome A10 when a 0.80 SNP index was used as the threshold. A total of 10 SNPs were involved in disrupting gene function. According to functions of genes, six SNPs located at the exonic or splicing site were selected for further analysis (Supplementary Table S4).

To confirm the candidate SNP, 93 F_2_ plants, including 48 recessive plants, were genotyped using KASP. We found that SNP 228 525 of *BraA10g000480.3C* co-segregated with the phenotype of the F_2_ plants. The mutant-phenotype plants exhibited a T:T genotype, whereas the wild-type-phenotype plants possessed a C:T or C:C genotype (Supplementary Table S5). Based on the above results, we hypothesized that *BraA10g000480.3C* was the canonical *BrAN* gene. *BrAN* is homologous to *AtAN* (*AT1G01510*), which is involved in controlling polar cell expansion in the leaf-width direction [22].

### Cloning and sequence analysis of *BrAN*

According to the MutMap method and KASP results, we subsequently cloned the DNA sequence and the cDNA sequence of *BrAN* gene from ‘FT’, *lhd1,* and *lhd2*. In ‘FT’, the DNA sequence of *BrAN* gene was 2 517 bp and consisted of seven exons and six introns; the cDNA sequence of *BrAN* gene was 1 896 bp and encoded a protein with 631 amino acid residues (Fig. 4b). The cDNA sequence of *Bran* gene in *lhd1* was 1 994 bp and encoded for a protein with 271 amino acid residues (Supplementary Figure S3a–c). Further sequence analysis showed that the inclusion of intron 2 in the *Bran* allele caused the difference between ‘FT’ and *lhd1*. Sequence analysis of the splice site established that an SNP (G to A) at position 842 on intron 2 led to intron retention. The splicing mutation caused a translational frameshift and a premature termination codon, thus producing a truncated product (Fig. 4c). The cDNA sequence of the *Bran* allele in *lhd2* was 1 896 bp and encoded a protein with 529 amino acid residues (Supplementary Figure S3d–f). Similarly, the sequencing results showed that the *Bran* allele in *lhd2* exhibited an SNP (G to A) at position 1 855 on exon 4, resulting in premature translation termination (Fig. 4d). The results suggested that the mutation phenotypes of *lhd1* and *lhd2* were controlled by the allelic gene, consistent with phenotypic observations in the allelism test between *lhd1* and *lhd2*. Moreover, comparative sequencing analysis showed no differences in the promoter sequence between ‘FT’ and the two allelic mutants (Supplementary Figure S4).

### Structural and phylogenetic analyses of BrAN

In ‘FT’, the BrAN protein possessed 631 amino acid residues with a molecular mass of 69.65 kDa and a pI of 6.32. Previous studies have reported that the AN protein shows substantial homology to CtBPs in animals. It is possible that AN protein in plants shares evolutionarily conserved functions with animal CtBPs. To understand the phylogenetic relationships of AN or CtBP proteins among various species and the functional relationship of BrAN, we aligned AN and CtBP proteins of several animal and plant species and constructed a phylogenetic tree, revealing that the proteins were divided into two subfamilies, named AN in plants and CtBP in animals (Supplementary Figure S5). The NAD-dependent 2-hydroxy acid dehydrogenase domain was conserved in all proteins. The LxCxE/D and nuclear localization signal motifs were present in plant AN but not present in animal CtBP, and the GxGxxG motif was present in CtBP but not in AN (Supplementary Figure S6). The amino acid sequence similarity of BrAN to AtAN, IAN, OsAN, ZmAN, LgAN, and MAN was 90.77%, 66.87%, 58.90%,57.84%, 58.05%, and 47.25%. BrAN was clustered with dicotyledons and closely related to AtAN in *Arabidopsis*, indicating that BrAN could have a key role in regulating leaf width by controlling cortical microtubule arrangement and epidermal cell shape in Chinese cabbage.

### Expression of *BrAN* in Chinese cabbage

**Figure 5:**
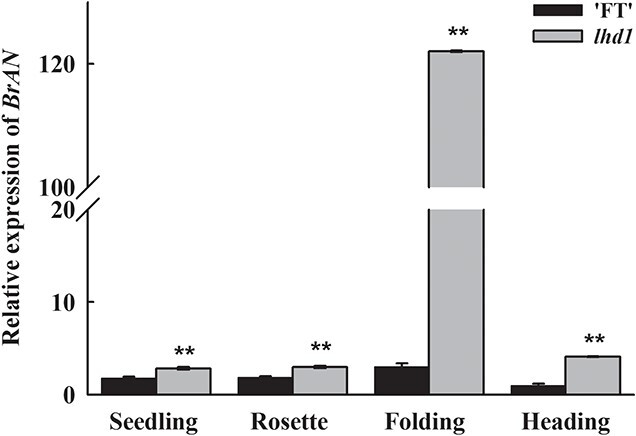
Expression levels of *BrAN* at various developmental stages. *BrActin* was used as an internal control. Bars show SD from three biological replicates, asterisks indicate statistical significance (Student’s *t*-test, ^**^P < 0.01).

The expression of *BrAN* at the seedling, rosette, folding, and heading stages were examined in both ‘FT’ and *lhd1* using quantitative real-time PCR to determine its physiological function. As shown in Fig. 5, *BrAN* was expressed in all four stages. Additionally, the expression of *BrAN* in *lhd1* was higher than in ‘FT’ and was highest at the folding stage.

### Transgenic functional verification of *BrAN*

**Figure 6:**
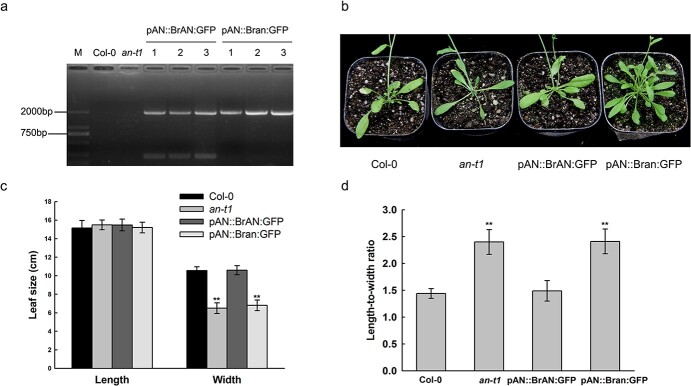
Transgenic functional verification of *BrAN.* (**a**) Identification of the transgenic lines by PCR. Amplification of the cDNA sequence of *BrAN* or *Bran* in *Arabidopsis.* M, molecular size markers; pAN::BrAN:GFP, pAN::BrAN:GFP plants; pAN::Bran:GFP, pAN::Bran:GFP plants. (**b**) Morphology of 3-week-old Col-0, *an-t1*, pAN::BrAN:GFP plants, and pAN::Bran:GFP plants. (**c**, **d**) Measurement and statistical analysis of leaf length and width (**c**), length/width ratio (**d**) of Col-0, *an-t1*, pAN::BrAN:GFP plants and pAN::Bran:GFP plants. Values are the means ± SD (n = 30 seedlings, ^**^P < 0.01). Statistically significant differences were calculated on the basis of Student’s *t*-tests.

**Figure 7:**
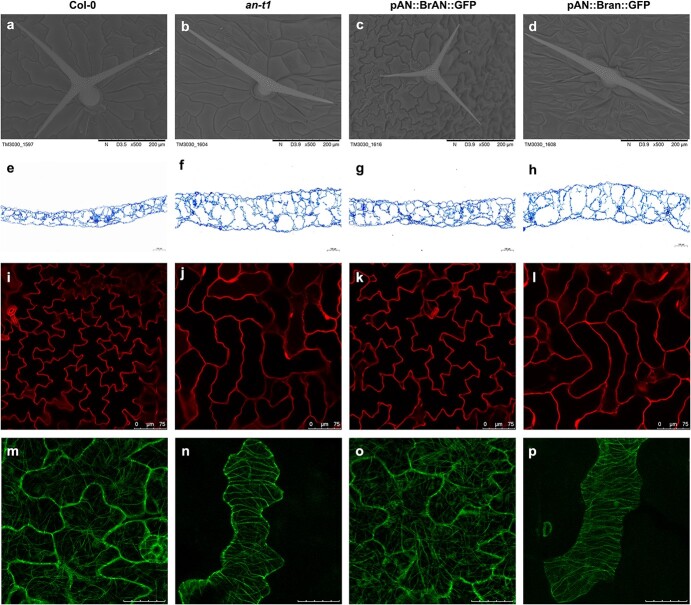
Cortical microtubule arrangement and pavement cell shape determined by *BrAN*. (**a**-**d**) SEM images of upper developing leaves, showing a mature trichome with three branches in Col-0 (**a**) and pAN::BrAN:GFP plants (**c**), and two branches in *an-t1* plants (**b**) and pAN::Bran:GFP plants (**d**). Scale bar = 200 μm. (**e**–**h**) Transverse sections of the leaf in Col-0 (**e**), *an-t1* (**f**) and pAN::BrAN:GFP plants (**g**), and pAN::Bran:GFP plants (**h**). Scale bar = 100 μm. (**i**–**l**) Pavement cells in Col-0 (**i**), *an-t1* (**j**), pAN::BrAN:GFP plants (**k**), and pAN::Bran:GFP plants (**l**). Scale bar = 75 μm. (**m**–**p**) Cortical microtubule arrangement in pavement cells in leaf-width direction of Col-0 (**m**), *an-t1* (**n**), and pAN::BrAN:GFP plants (**o**), and pAN::Bran:GFP plants (**p**). Scale bar = 25 μm.

The *Arabidopsis an* mutant exhibited narrower and thicker leaves than Col-0 due to a defect in the polarity-specific expansion of cells in the leaf-width direction. *an-t1* (SALK_026489C), the T-DNA insertional mutant, was confirmed to be a null mutant with loss of *AtAN* function [29]. To prove the function of *BrAN*, we generated plants by driving *BrAN* and *Bran* from the pAN promoter in *an-t1* background, respectively. Transgenic lines were identified by PCR using the primers for *BrAN* cDNA cloning. Homozygous T3-positive lines were used for further analyses (Fig. 6a). Based on the results of this transformation, *BrAN* was functional in *Arabidopsis*. First, there were no significant morphological differences between Col-0 and transgenic plants expressing *BrAN* (pAN::BrAN:GFP plants) (Fig. 6b). The narrow leaf phenotype of *an-t1* was completely rescued by pAN::BrAN:GFP, but leaves were still narrow in transgenic plants expressing *Bran* (pAN::Bran:GFP plants) (Fig. 6c, d). Second, the number of trichome branches was recovered from two in *an-t1* to three in pAN::BrAN:GFP plants (Fig. 7a–d). Third, polar changes in cell morphology in *an-t1* were not observed in pAN::BrAN:GFP plants (Fig. 7e, g), and the leaf thickness of pAN::Bran:GFP plants was similar to that of *an-t1* (Fig. 7f, h). Moreover, the epidermal cells of Col-0 were in a typical jigsaw puzzle shape and pAN::BrAN:GFP plants showed the shape of the puzzle (Fig. 7i, k), pAN::Bran:GFP plants did not rescue the simple shape of cells (Fig. 7j, l). These results confirmed the role of *BrAN* in epidermal cell shape and leaf width.

### Observation results of cortical microtubule arrangement

The polarity of cells is closely related to the orientation of cellulose microfibrils, which is governed by cortical microtubule arrangement [26]. We used *Arabidopsis* as a model to analyse the orientation of cortical microtubules and function of *BrAN* because it was technically impossible to observe microtubules in Chinese cabbage cells. Therefore, the cortical microtubule arrangement in Col-0, *an-t1,* pAN::BrAN:GFP, and pAN::Bran:GFP plants leaf cells was examined. As shown in Fig. 7n, cortical microtubules in *an-t1* pavement cells tended to be distributed parallel to the leaf-width direction. In Col-0 pavement cells, the cortical microtubules were randomly distributed in most directions within the cells, and their arrangement in pAN::BrAN:GFP plants was similar to that in Col-0 (Fig. 7m, o). However, transformation of *Bran* did not rescue the abnormal arrangement in *an-t1* (Fig. 7p)*.*

## Discussion

In this study, two Chinese cabbage leafy heading-deficient allelic mutants, *lhd1* and *lhd2*, were selected to explore the genetic mechanism underlying leafy head development. Several evidences suggested that *BrAN* was responsible for the mutant phenotype. An SNP (G to A) at the splicing site on the second intron led to intron retention and premature translation termination of *BrAN* in *lhd1*, and an SNP (G to A) on the fourth exon in *BrAN* led to premature translation termination in *lhd2*. Transgenic *BrAN* was functional in *Arabidopsis an-t1,* rescuing the mutant phenotype, which showed features including narrow leaves, defects in cell polarity, and abnormal arrangement of cortical microtubules. Transgenic *Bran* did not rescue the mutant phenotype. These results strongly suggested that *BrAN* contributed to leafy head formation by regulating leaf width in Chinese cabbage, providing a novel target gene to unravel the molecular mechanisms of leafy head development.

Leafy head is an important agronomic trait of Chinese cabbage. Some progress has been made in determining the genetic mechanisms of leafy head development [8, 33–35]. Mutagenesis is an important method for functional genome studies, and numerous genetically diversified mutants can be obtained by mutagenesis, some of which might be allelic mutants, which originate from same gene mutations at different sites. Because transgenic technology has not yet been perfected for Chinese cabbage functional verification, allelic mutants are ideal materials for verification of gene function [36, 37]. Our study used two allelic leafy heading-deficient mutants to verify gene function, instead of transformation of Chinese cabbage, and demonstrated that the leafy heading-deficient phenotype in *lhd1* and *lhd2* was controlled by the allelic gene *BrAN*.

Leaf morphology is of particular significance for leafy head development in Chinese cabbage. At the rosette stage, the leaves grow large and rounded and begin to fold upward. At the folding stage, the leaves curve inward to form an internal mold for further head development. Rosette leaf width largely determines heading degree and size which is increased following the head formation process [8]. Therefore, leaf incurvature is essential to leafy head formation [9], and leaf width is important for leaf overlapping and tight arrangement. During all growth stages, the leaf width of leafy heading-deficient mutants was significantly less than that of ‘FT’, whereas the leaf length of leafy heading-deficient mutants was similar to that of ‘FT’ (Table 1; Supplementary Table S1). Cellular characteristics, such as the size and shape of cells, as well as the extent and orientation of cell division and expansion, determine leaf morphology [16]. The shape of leafy heading-deficient mutant epidermal cells was simpler than that of ‘FT’ (Fig. 3a, b; Supplementary Fig. S2a, b). The cellular organization of palisade and spongy parenchyma and thickness of leaves in ‘FT’ and two mutants were different (Fig. 3c–f; Supplementary Fig. S3c–f). Further observation of cortical microtubules showed that the cell polarity change was caused by increased regularity of cortical microtubule alignment parallel to the leaf-width direction (Fig. 7n), consistent with *Arabidopsis* [22, 26]. We proposed that due to a change in cell polarity caused by the changes in microtubule arrangement triggered by the *BrAN* mutation, the cells growing originally along the width of the leaves grew along the thickness of the leaves (Fig. 3c–f; Supplementary Fig. S3c–f). Thus the leaves of *lhd1* and *lhd2* became narrow and thick, restricting leafy head formation, which is based on wide leaves. Simultaneously, we noted that the leaves did not develop an inward curvature but expanded outward at the late vegetative growth stage in *lhd1* and *lhd2*. The leaf adaxial–abaxial pattern in ‘FT’ changed at the heading stage compared with the rosette stage, which did not occur in *lhd1* and *lhd2* (Fig. 3e, f; Supplementary Figure S3e, f). Therefore, we hypothesized that the shape and arrangement of cells might influence the inward curvature of *lhd1* and *lhd2* leaves, consistent with previous study findings [38]. Additionally, from the folding stage, the angle between the leaves and the ground in *lhd1* was smaller than that of ‘FT’, which could influence leafy head formation. These hypotheses need to be tested further.

Some advances have been made regarding AN, which is a plant homolog of animal CtBP. AN functions have been reported in plant species, including AtAN in *Arabidopsis*, IAN in Japanese morning glory (*Ipomoea nil* L. Roth), LgAN in Dahurian larch (*Larix gmelinii*), and MAN in liverwort (*Marchantia polymorpha*) [22, 39–41]. The sequence similarity between BrAN and its homologs suggested that the molecular function is conserved in different plants (Supplementary Fig. S6). Similar to *an* mutants in *Arabidopsis* [23], narrow and thick leaves, narrow petals, less complex epidermal cells and more regularly arranged cortical microtubules were observed in the leafy heading-deficient mutants. Our study is the first to report the function of *BrAN* in Chinese cabbage leafy head formation. Interestingly, *BrAN* was significantly upregulated in mutant and expressed highest at the folding stage (Fig. 5), although there were no differences in the promoter sequence between WT and mutants (Supplementary Fig. S4). There were more leaves in pAN::Bran:GFP plants than pAN::BrAN:GFP plants (Fig. 6b), consistent with the results that *lhd* mutants had more leaves than ‘FT’ at the mature stage (Fig. 2d), suggesting that a mutated allele *Bran* might have function in increasing the number of leaves. It would be fascinating to find out what induces the expression of *BrAN* and the function of mutated *BrAN*.

Previous studies have shown that AN is involved in the morphological development of leaves and floral organs by controlling cortical microtubule arrangement [24, 26]. AN and ZWICHEL, a kinesin motor molecule involved in leaf trichome branching by controlling the microtubule cytoskeleton, interact genetically and physically [25]. However, AN is not a microtubule-associated protein, and it localizes to punctuate structures around the Golgi rather than cortical microtubules [41]. Cellulose synthase complexes are assembled in the Golgi and then transported to the plasma membrane by vesicles to synthesize cellulose. Cortical microtubule paths determine the orientation of cellulose microfibrils [42]. Therefore, the arrangement of cortical microtubules is involved in cellulose synthase transportation and affects the cellulose microfibrils deposition, which further influences the anisotropic growth of plant cells. Several differentially expressed tubulin genes were identified, and the morphological differences related to leaf cell development or organization were studied in heading Chinese cabbage and non-heading pak choi (*B. rapa* subsp*. chinensis*) [38]. As expected, the cortical microtubules in *an-t1* were more regularly and orderly arranged along the leaf-width direction than those in the Col-0 and pAN::BrAN:GFP plants (Fig. 7m–p). The polarity of cell morphology changed in *an-t1*, and it was restored in pAN::BrAN:GFP plants (Fig. 7e–h). These results indicated that *BrAN* plays a significant role in cortical microtubule arrangement and epidermal cell shape in the development of Chinese cabbage leaves.

Our study demonstrated that abnormal microtubule arrangements and epidermal cell shape caused by mutations in *BrAN* led to the narrow leaves, which couldn’t meet the prerequisite, wide leaves, for leafy head formation in mutants. These findings expand our understanding of the mechanism underlying leafy head formation and provide a novel target gene to explore the genetic mechanism underlying leafy head development in Chinese cabbage.

## Materials and methods

### Plant materials and growth conditions

The wild-type Chinese cabbage used was the double haploid line, ‘FT’ derived from ‘Fukuda 50’ with an ovoid leafy head. Several leafy heading-deficient mutant plants were obtained by immersing ‘FT’ germinated seeds in 0.8% ethyl methanesulfonate. We selected two leafy heading-deficient mutants, *lhd1* and *lhd2*, for further study. The plants were grown in a plastic greenhouse at Shenyang Agricultural University, Shenyang, China, in 2019.

The *Arabidopsis* plants used in this study were from a Columbia background. The wild-type (Col-0) and *GFP-Tubulin* (*GFP-TUA*; a microtube maker line) were preserved in Shenyang Agricultural University, and *an-t1* was obtained from AraShare (Fujian, China). The plants were grown in a growth chamber at 22°C under a 16 h light/8 h dark cycle and 80% relative humidity.

### Genetic analysis

For genetic analysis, *lhd1* was crossed with ‘FT’ to develop F_1_, BC_1_, and F_2_ populations, and the F_2_ population was used for genetic mapping and genotyping. We used the same strategy to generate the *lhd2* populations. The segregation ratio of the BC_1_ and F_2_ populations was analysed using the Chi-square (*χ*^2^) test.

### Allelism test between *lhd1* and *lhd2*

For the allelism test, we crossed *lhd1* × ‘FT’ (*lhd1* F_1_) and *lhd2* × ‘FT’ (*lhd2* F_1_) to determine whether the mutated genes in *lhd1* and *lhd2* were allelic because of the lower seed setting rate in the F_1_ hybrids *lhd1* × *lhd2* and *lhd2* × *lhd1*. Phenotypic data were collected from *lhd1* F_1_ × *lhd2* F_1_ and calculated to obtain the separation ratio.

### Measurement of leaf parameters and association analysis

To characterize the difference in leaf morphology between the ‘FT’ and two leafy heading-deficient mutants, the length (including the petiole), and width (at the widest points) of the largest leaf at the seedling stage, fifth rosette leaves at the rosette stage, first folding leaves at the folding stage, and first head leaves at the heading stage were measured; the length-to-width ratio was then calculated. The leaves were selected in the same order starting from the exterior of the ‘FT’ and leafy heading-deficient mutants.

All measurements, including the leaf index, trichomes, and pavement cell morphology were made on the fifth leaf of 4-week-old *Arabidopsis* plants [24].

### Cytological observation of leaf-width direction

To identify whether the narrow leaf phenotype was related to the change in cell morphology in the leaf-width direction, cross-sections of the fifth rosette and first head leaves of ‘FT’ and leafy heading-deficient mutants were observed. The fifth leaves of the Col-0, *an-t1*, and transgenic plants expressing *BrAN* (pAN::BrAN:GFP plants) were sampled at 4 weeks. The samples were treated as previously described [43]. The leaf sections were observed under an optical microscope (Leica Microsystems, Wetzlar, Germany).

### Candidate gene identification using MutMap

A modified MutMap method was applied to a fine map to identify candidate genes for *BrAN* [44]. Thirty leafy heading-deficient plants in *lhd1* × ‘FT’ F_2_ were selected to form a leafy heading-deficient mutant bulk pool (D-pool). DNA from the ‘FT’, *lhd1,* and D-pool was isolated using a plant DNA extraction kit (Tiangen, Beijing, China) and was re-sequenced using a NovaSeq 6000 sequencer (Illumina, San Diego, CA, USA). Clean reads were obtained using the NGSQC toolkit (http://www.nipgr.res.in/ngsqctoolkit.html) after filtering reads as follows: (i) reads containing the adapter sequence; (ii) low-quality reads; and (iii) sequences with a base number less than 75 bp. Then the clean reads were aligned to the Chinese cabbage reference genome sequence using BWA software [45]. INDELs and SNPs were detected using GATK software [46]. Functional annotation was conducted using ANNOVAR software [47]. The mutation information was mapped to the genome using Circos software [48].

### SNP genotyping using KASP assay

KASP assay was used for SNP genotyping to detect the co-segregation of the SNPs and to confirm the candidate gene. For KASP genotyping, 93 F_2_ plants, including 48 plants without the leafy head and 45 plants with the leafy head were selected. The experiment was conducted by Institute of Vegetables and Flowers, Chinese Academy of Agricultural Sciences, Beijing, China. Allele-specific primers bearing the fluorescence probes FAM and HEX and the common primers used for KASP are listed in Supplementary Table S6.

### Gene cloning and expression analysis

For cloning and expression analysis, total RNA from leaves at the seedling, rosette, folding, and heading stages of ‘FT’ and two leafy heading-deficient mutants was extracted using an RNA extraction kit (Aidlab, Beijing, China). Total RNA was reverse transcribed to first-strand cDNA using a Fast Quant RT Kit (Tiangen, Beijing, China). Quantitative real-time PCR was performed using ChamQ Universal SYBR qPCR Master Mix (Vazyme, Nanjing, China) on QuantStudio™ 6 Flex Real-Time PCR System (Applied Biosystems, Waltham, MA, USA). To reduce errors, three biological and technical replications were conducted in all experiments. The *BrActin* (*BraA10g027990.3C*) gene in Chinese cabbage was used as the internal control [15]. The relative gene expression levels were calculated using the 2^-ΔΔCt^ method [49]. All primers used in this experiment are listed in Supplementary Table S7.

### AN protein sequence alignment and phylogenetic analysis

AN and CtBP protein sequences of the various species were obtained from the NCBI database (http://www.ncbi.nlm.nih.gov/BLAST) to analyse the phylogenetic relationships with Chinese cabbage AN protein in this study. The accession numbers of protein sequences and genus names of the 10 species used were as follows: *L. gmelinii* BAG68439, *I. nil* BAC58021, *M. polymorpha* BAC81145, *Oryza sativa Japonica Group* AAP54786, *Zea mays* NP_001151564, *Arabidopsis thaliana* NP_563629, *B. rapa* XP_009119703, *Homo sapiens* AAD14597, *Caenorhabditis elegans* CCG28183 and *Drosophila melanogaster* NP_001262524. The AN protein homologs were aligned using ClustalW 1.8, and a neighbor-joining tree was constructed using MEGA 5.1, based on a bootstrap of 1000 replicates.

### Vector construction and *Arabidopsis* transformation

To generate transgenic plants, pAN::BrAN:GFP and pAN::Bran:GFP were produced from pBWA(V)HII-osgfp-TONS. pAN::BrAN:GFP was a vector that contained a Gateway cassette followed by GFP gene, the 2000 bp genomic fragment upstream of the *BrAN* gene, and coding sequence from initiation codon to the codon before the stop codon in ‘FT’. *Bran* from *lhd1* was cloned to generate the pAN::Bran:GFP using the same strategy. The selected coding regions were amplified using primers (Supplementary Table S7). The transformation of *an-t1* mutants with the binary vector pAN::BrAN:GFP and pAN::Bran:GFP bearing a kanamycin resistance gene was performed using the floral infiltration method [50].

### Scanning electron microscopy (SEM)

To observe the trichome morphology, the mature trichome on fresh leaves was examined using the TM3030 scanning electron microscope (Hitachi, Tokyo, Japan), as previously described [51].

### Confocal microscopy

Fluorescence signal was examined using confocal laser scanning microscopy. For live confocal imaging of cortical microtubules, *GFP-TUA* was crossed to *an-t1*，pAN::BrAN:GFP and pAN::Bran:GFP plants respectively to generate *an-t1* and pAN::BrAN:GFP and pAN::Bran:GFP plants stably expressing *GFP-TUA*. Seven-day-old cotyledons were selected for mesophyll cell samples. GFP was excited at a wavelength of 488 nm and emission was measured at a wavelength of 500–550 nm.

For pavement cell morphology analysis, propidium iodide was applied to cells, and the samples were imaged with an excitation wavelength of 561 nm and emission wavelength of 580–630 nm. The parameters cell area and number of cells were measured by ImageJ software.

## Acknowledgements

This study was supported by the National Natural Science Foundation of China (32002033), Liaoning Province Scientific Research Funding Project (LSNQN202019) and Graduate Student Innovation Cultivation Project of Shenyang Agricultural University (2021YCXB16). We would like to thank Editage (www.editage.cn) for their assistance with English language editing.

## Author contributions

H.F., C.W., and Y.X. designed the experiments. Y.X. conducted the experiments and wrote the manuscript. C.T. and Y.X. performed data analyses. S.H., Y.G., and Z.L. helped generate the mutant. N.W., L.W., and Y.W. participated in the laboratory experiments. H.F. and C.T. revised the manuscript. All the authors read and approved the final manuscript.

## Data availability

Sequencing data of ‘FT’, *lhd1*, and F_2_ population were deposited in the SRA Database in NCBI (Accession number: SRR15174650, SRR19629893 and SRR19629892). Other data supporting our findings are available in the manuscript file or from the corresponding author upon request.

## Conflict of interest

The authors declare no conflict of interest.

## Supplementary data

Supplementary data is available at *Horticulture Research* online.

## Supplementary Material

Web_Material_uhac167
