# Supplementary material for: BrAN contributes to leafy head formation by regulating leaf width in Chinese cabbage (Brassica rapa L. ssp. pekinensis)
Source: Hortic Res. 2022 Jul 27;9:uhac167. doi: 10.1093/hr/uhac167 (PMC9531340; doi:10.1093/hr/uhac167)
Supplement: Web_Material_uhac167 [file web_material_uhac167.docx]

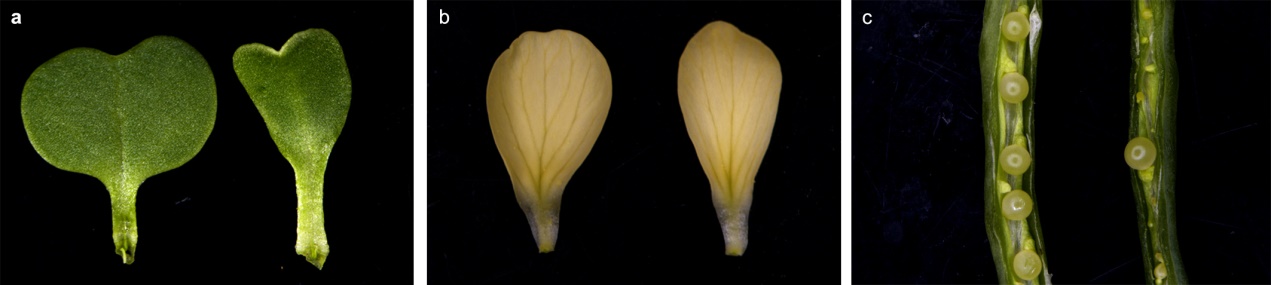


**Fig. S1. Cotyledons (a), petals (b) and seeds in the pod (c) of ‘FT’ (left) and *lhd* mutant (right).**


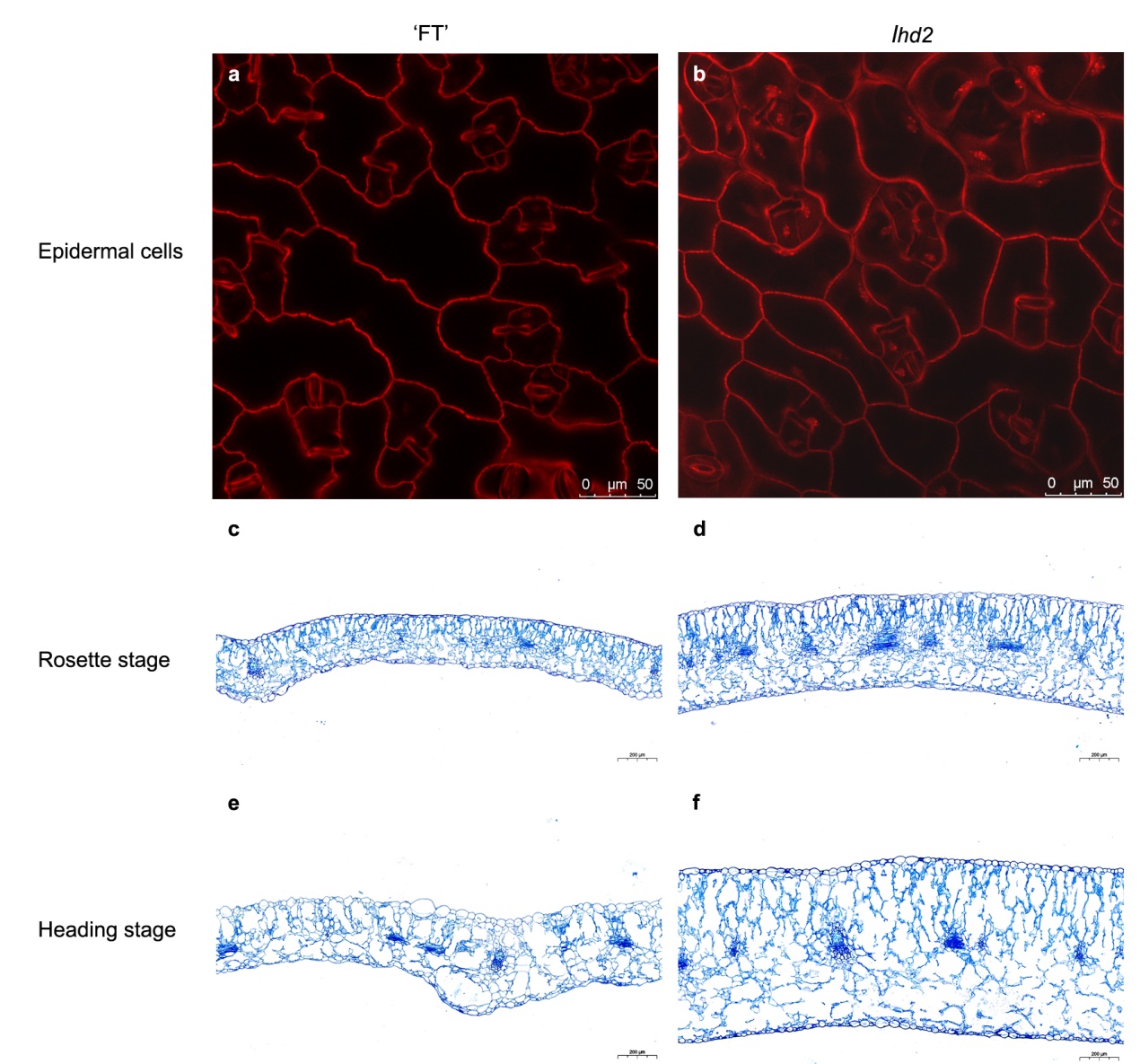


**Fig. S2. Cell phenotypes of wild-type ‘FT’ and *lhd2.*** Leaf abaxial epidermal cells of ‘FT’ (a) and *lhd2* (b) seedling leaf. Scale bar = 50 μm. Leaf transverse sections of ‘FT’ (c) and *lhd2* (d) rosette leaf. Scale bar = 200 μm. Leaf transverse sections of ‘FT’ (e) and *lhd2* (f) heading leaf. Scale bar = 200 μm.


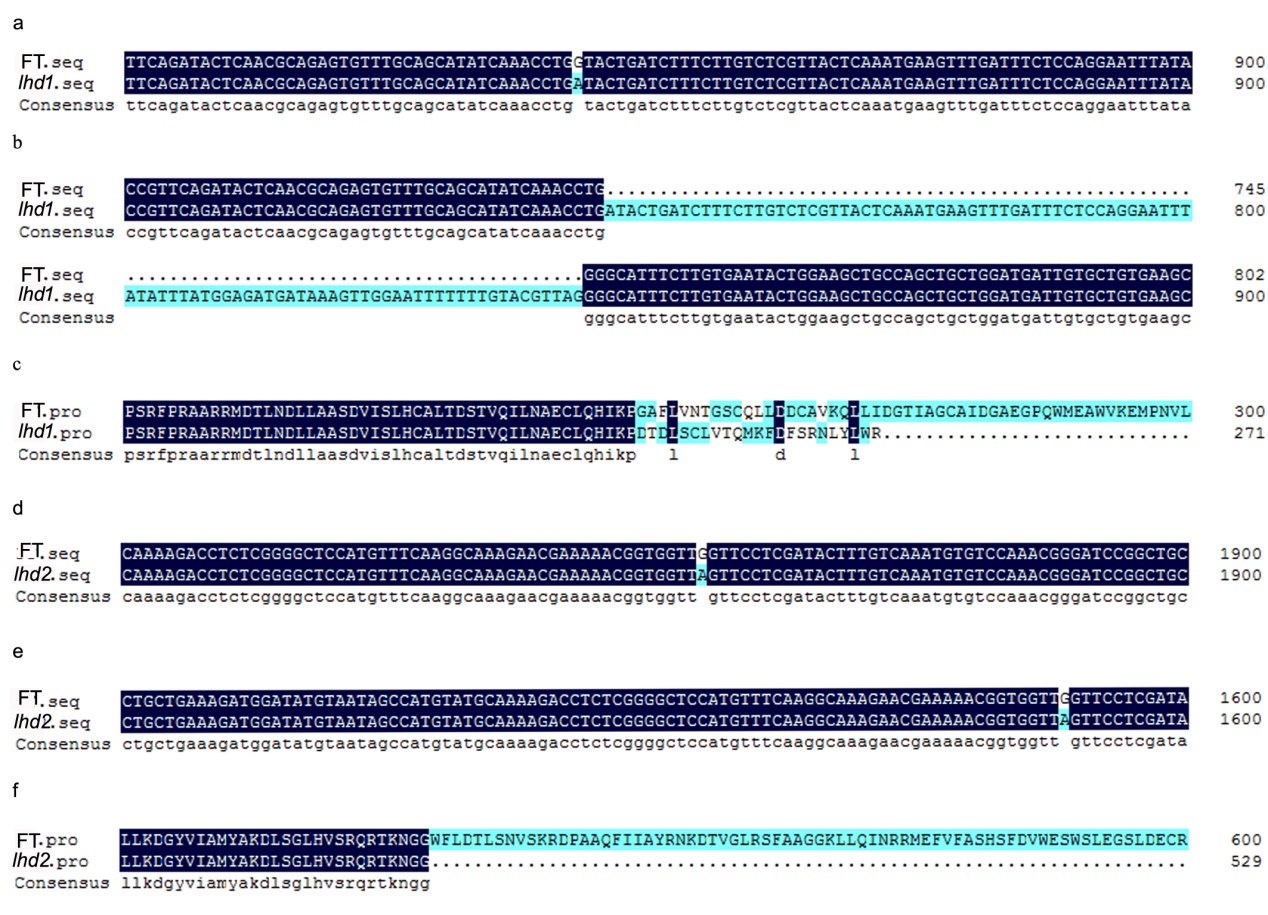


**Fig. S3. Sequence analyses of *BraA10g000480.3C.*** Comparison of genomic sequence (a), coding sequence (b) and amino acid sequence (c) between ‘FT’ and *lhd1*. Comparison of genomic sequence (d), coding sequence (e) and amino acid sequence (f) between ‘FT’ and *lhd2*.

**
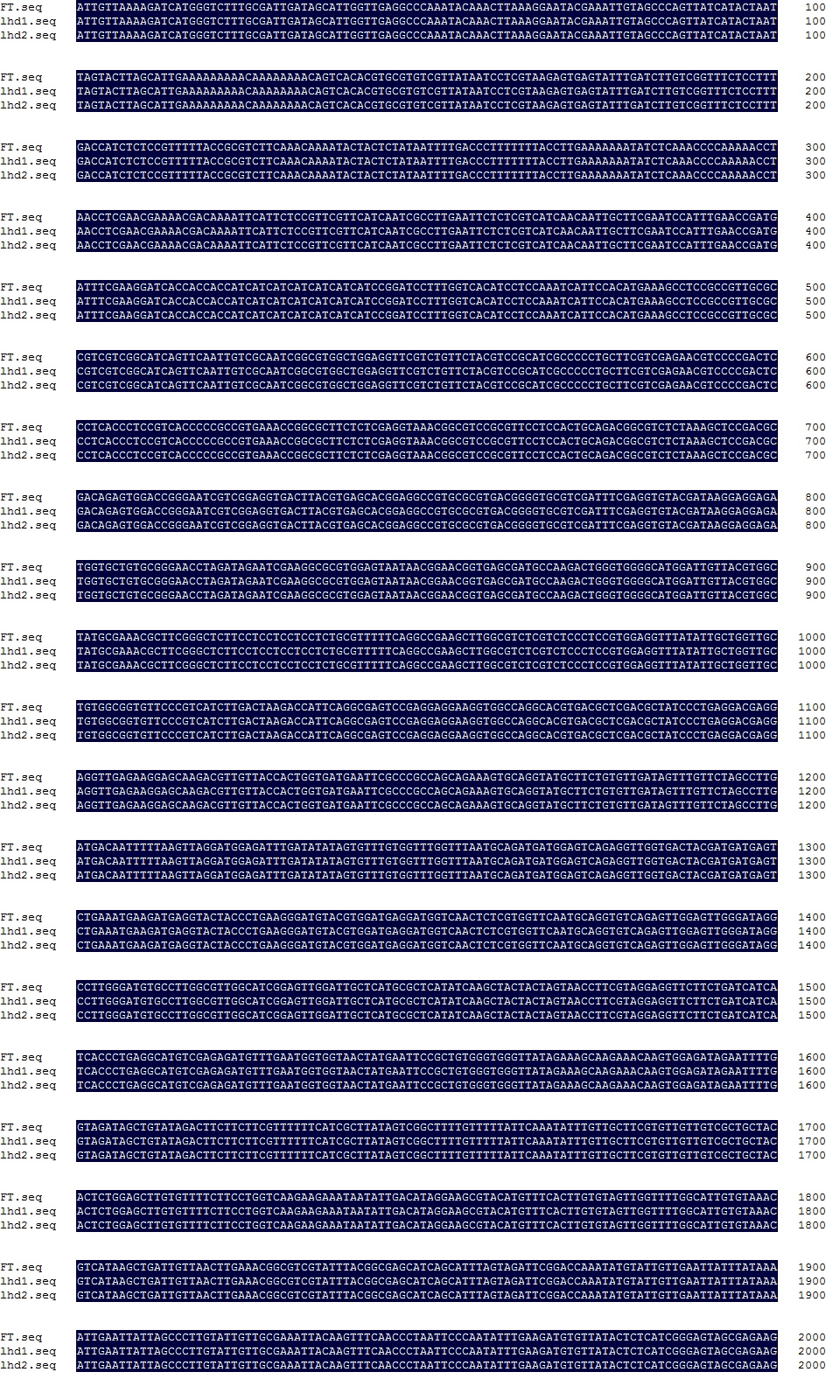
**

**Fig. S4. Comparison of the promoter sequence*.***


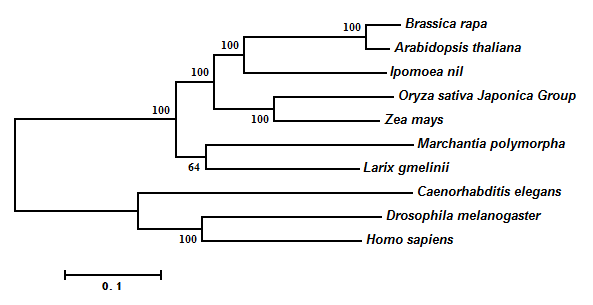


**Fig. S5 Phylogenetic tree of AN and CtBP proteins.** The neighbor-joining tree was constructed using MEGA 6.0, based on a bootstrap of 1,000 replicates. Numbers on the tree represented bootstrap values. Scale bar represented branch length.


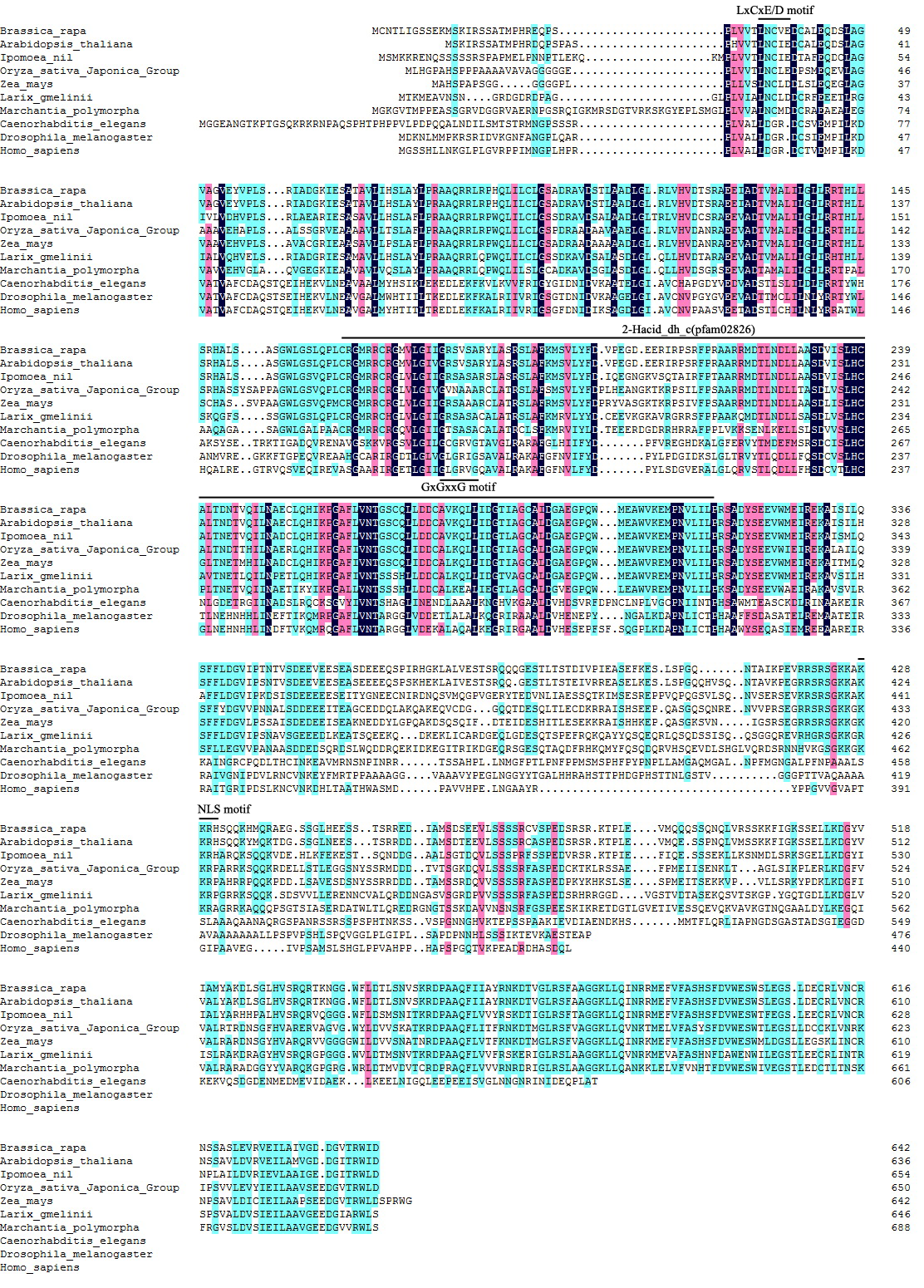


**Fig. S6 Amino acid alignment of different AN proteins in plants and CtBPs from several representative species.** Sequence alignments were performed using DNAMAN software. Residues with 100% similarity are shaded in black. Those with ≥75% similarity are shaded in pink. Those with ≥50% similarity are shaded in blue.

**Table S1 Parameters of leaves in ‘FT’ and *lhd2***

The parameters length, width, and length-to-width ratio were measured at four different stages. Values are the means ± SD (n=20 seedlings, ***P*<0.01). Statistically significant differences were calculated based on Student’s *t*-tests.

| Plant | Length (cm) | Width (cm) | Length-to-width Ratio |
| --- | --- | --- | --- |
| Seedling Stage |  |  |  |
| ‘FT’ | 7.07±0.43 | 4.00±0.35 | 1.78±0.13 |
| *lhd2* | 7.13±0.43 | 2.46±0.32** | 2.94±0.27** |
| Rosette Stage |  |  |  |
| ‘FT’ | 12.68±0.21 | 8.83±0.15 | 1.44±0.02 |
| *lhd2* | 12.92±0.20 | 6.66±0.14** | 1.95±0.02** |
| Folding Stage |  |  |  |
| ‘FT’ | 19.00±0.97 | 18.96±1.94 | 1.01±0.08 |
| *lhd2* | 19.25±1.00 | 14.53±0.98** | 1.32±0.08** |
| Heading Stage |  |  |  |
| ‘FT’ | 25.00±0.68 | 22.34±0.67 | 1.12±0.04 |
| *lhd2* | 25.26±0.80 | 17.18±0.63** | 1.47±0.16** |

**Table S2 Adaxial epidermal cell size and number of** **‘FT’, *lhd1* and *lhd2***

The parameters cell area, number of cells were measured from seedlings leaves by ImageJ software. Values are the means ± SD (n=5 seedlings, **P<0.01). Statistically significant differences were calculated based on Student’s t-tests.

| Plant | Area (µm^2^) | Number of cells |
| --- | --- | --- |
| ‘FT’ | 3067.58±1827.01 | 4756.84±421.11 |
| *lhd1* | 1611.98±709.68** | 3349.24±339.70** |
| *lhd2* | 1705.97±719.76** | 3414.56±441.02** |

**Table S3 Genetic analysis of leafy heading-deficient phenotype in mutant *lhd2***

| Generations | Total | ‘FT’ | *lhd2* | Segregation Ratio | χ2 |
| --- | --- | --- | --- | --- | --- |
| P_1_(‘FT’) | 50 | 50 | 0 |  |  |
| P_2_(*lhd2*) | 25 | 0 | 25 |  |  |
| F_1_(P_1_×P_2_) | 50 | 50 | 0 |  |  |
| F_1_(P_2_×P_1_) | 22 | 22 | 0 |  |  |
| BC_1_(F_1_×‘FT’) | 48 | 48 | 0 |  |  |
| BC_1_(F_1_×*lhd2*) | 50 | 26 | 24 | 1.083:1 | 0.020 |
| F_2_ | 780 | 600 | 180 | 3.333:1 | 1.438 |

**Table S4 List of candidate SNPs on chromosome A10**

| Pos | Ref | WT | Mut | SNP  _index | Location | Gene | Exon  ID | Nucleotide  change | Amino acid  change | Mutation type | Function annotation |
| --- | --- | --- | --- | --- | --- | --- | --- | --- | --- | --- | --- |
| 228525 | C | C | T | 1.0000 | splicing | *BraA10g000480.3C* | -- | -- | -- | -- | Control polar cell expansion in the leaf width direction |
| 1333570 | C | C | T | 1.0000 | exonic | *BraA10g002600.3C* | exon1 | c.C62T | p.P21L | nonsynonymous SNV | Vacuolar sorting-associated protein |
| 1968210 | C | C | T | 0.8750 | exonic | *BraA10g003740.3C* | exon7 | c.G1237A | p.D413N | nonsynonymous SNV | Helicase protein with RING/U-box domain-containing protein |
| 2329979 | C | C | T | 0.8286 | exonic | *BraA10g004420.3C* | exon6 | c.C1435T | p.P479S | nonsynonymous SNV | Encodes a gamma-secretase subunit |
| 2935902 | C | C | T | 0.8462 | exonic | *BraA10g005490.3C* | exon1 | c.G719A | p.G240E | nonsynonymous SNV | Alpha/beta-Hydrolases superfamily protein |
| 4257994 | C | C | T | 0.8947 | exonic | *BraA10g007150.3C* | exon4 | c.G1990A | p.V664I | nonsynonymous SNV | Involved in lipid metabolic process |
| 4886731 | C | C | T | 0.8571 | exonic | *BraA10g007580.3C* | exon1 | c.G208A | p.E70K | nonsynonymous SNV | Unknown |
| 5436285 | C | C | T | 0.8400 | exonic | *BraA10g008030.3C* | exon3 | c.C331T | p.L111F | nonsynonymous SNV | UDP-Glycosyltransferase superfamily protein |
| 7754665 | C | C | T | 0.9583 | exonic | *BraA10g009700.3C* | exon2 | c.G489A | p.W163X | stopgain | Unknown |
| 8431726 | C | C | T | 0.8000 | exonic | *BraA10g010270.3C* | exon2 | c.G128A | p.R43H | nonsynonymous SNV | Microtubule associated protein (MAP65/ASE1) family protein |

**Table S5 Genotyping results of SNPs**

| F_2_ population | Phenotype | Genotype-SNP 228525 | Genotype-SNP 2329979 | Genotype-SNP 4886731 | Genotype-SNP 5436285 | Genotype-SNP 7754665 | Genotype-SNP 8431726 |
| --- | --- | --- | --- | --- | --- | --- | --- |
| 1 | mutant | T:T | T:T | C:T | C:T | C:T | C:T |
| 2 | mutant | T:T | T:T | T:T | C:T | T:T | C:C |
| 3 | mutant | T:T | C:T | C:T | C:T | C:T | C:T |
| 4 | mutant | T:T | T:T | T:T | T:T | T:T | T:T |
| 5 | mutant | T:T | T:T | T:T | T:T | T:T | T:T |
| 6 | mutant | T:T | T:T | T:T | T:T | T:T | T:T |
| 7 | mutant | T:T | T:T | T:T | T:T | T:T | T:T |
| 8 | mutant | T:T | T:T | T:T | T:T | T:T | T:T |
| 9 | mutant | T:T | T:T | T:T | T:T | T:T | T:T |
| 10 | mutant | T:T | T:T | C:T | C:T | C:T | C:T |
| 11 | mutant | T:T | T:T | T:T | T:T | T:T | T:T |
| 12 | mutant | T:T | C:T | C:T | C:T | C:T | C:T |
| 13 | mutant | T:T | T:T | T:T | T:T | T:T | T:T |
| 14 | mutant | T:T | T:T | C:T | C:T | C:T | C:T |
| 15 | mutant | T:T | T:T | T:T | T:T | C:C | C:C |
| 16 | mutant | T:T | T:T | T:T | T:T | T:T | T:T |
| 17 | mutant | T:T | T:T | T:T | T:T | T:T | T:T |
| 18 | mutant | T:T | T:T | C:T | C:T | C:T | C:T |
| 19 | mutant | T:T | C:T | C:T | C:T | C:T | C:T |
| 20 | mutant | T:T | T:T | T:T | T:T | T:T | T:T |
| 21 | mutant | T:T | T:T | T:T | T:T | T:T | T:T |
| 22 | mutant | T:T | C:T | C:T | C:T | C:T | C:T |
| 23 | mutant | T:T | T:T | T:T | T:T | T:T | T:T |
| 24 | mutant | T:T | C:T | C:T | C:T | C:T | C:T |
| 25 | mutant | T:T | T:T | T:T | T:T | T:T | T:T |
| 26 | mutant | T:T | T:T | C:T | C:T | C:T | C:T |
| 27 | mutant | T:T | T:T | T:T | T:T | T:T | T:T |
| 28 | mutant | T:T | C:T | C:T | C:T | C:T | C:T |
| 29 | mutant | T:T | T:T | T:T | T:T | T:T | T:T |
| 30 | mutant | T:T | T:T | T:T | T:T | T:T | T:T |
| 31 | mutant | T:T | T:T | C:T | C:T | C:T | C:T |
| 32 | mutant | T:T | T:T | T:T | T:T | T:T | T:T |
| 33 | mutant | T:T | T:T | T:T | T:T | T:T | T:T |
| 34 | mutant | T:T | C:T | C:T | C:T | C:T | C:T |
| 35 | mutant | T:T | T:T | T:T | T:T | T:T | T:T |
| 36 | mutant | T:T | T:T | T:T | T:T | T:T | T:T |
| 37 | mutant | T:T | C:T | C:T | C:T | C:T | C:T |
| 38 | mutant | T:T | T:T | T:T | T:T | T:T | T:T |
| 39 | mutant | T:T | C:T | C:C | C:C | C:C | C:C |
| 40 | mutant | T:T | T:T | C:T | C:T | C:T | C:T |
| 41 | mutant | T:T | T:T | T:T | T:T | T:T | T:T |
| 42 | mutant | T:T | T:T | T:T | T:T | T:T | T:T |
| 43 | mutant | T:T | T:T | T:T | T:T | T:T | T:T |
| 44 | mutant | T:T | T:T | T:T | T:T | T:T | T:T |
| 45 | mutant | T:T | T:T | T:T | T:T | T:T | T:T |
| 46 | mutant | T:T | C:T | C:T | C:T | C:T | C:T |
| 47 | mutant | T:T | T:T | T:T | T:T | T:T | C:T |
| 48 | mutant | T:T | T:T | T:T | T:T | T:T | C:T |
| 49 | wild-type | C:T | C:T | C:T | C:T | C:T | C:T |
| 50 | wild-type | C:T | C:T | C:T | C:T | C:T | C:T |
| 51 | wild-type | C:T | C:T | C:T | C:T | C:T | C:T |
| 52 | wild-type | C:T | C:T | C:T | C:T | C:T | C:T |
| 53 | wild-type | C:T | C:T | C:T | C:T | C:T | C:T |
| 54 | wild-type | C:C | C:T | C:T | C:T | C:T | C:T |
| 55 | wild-type | C:T | C:T | C:T | C:T | C:T | C:T |
| 56 | wild-type | C:T | C:T | C:T | C:T | C:T | C:T |
| 57 | wild-type | C:T | C:T | C:T | C:T | C:T | C:T |
| 58 | wild-type | C:T | C:T | C:T | C:T | C:T | C:T |
| 59 | wild-type | C:T | C:T | C:T | C:T | C:T | C:T |
| 60 | wild-type | C:T | C:T | C:T | C:T | C:T | C:T |
| 61 | wild-type | C:C | C:C | C:C | C:C | C:C | C:C |
| 62 | wild-type | C:T | C:T | C:T | C:T | C:T | C:T |
| 63 | wild-type | C:T | C:C | C:C | C:C | C:C | C:C |
| 64 | wild-type | C:T | C:T | C:T | C:T | C:T | C:T |
| 65 | wild-type | C:T | C:T | C:T | C:T | C:T | C:T |
| 66 | wild-type | C:T | C:T | C:T | C:T | C:T | C:T |
| 67 | wild-type | C:C | C:C | C:C | C:C | C:C | C:C |
| 68 | wild-type | C:C | C:C | C:C | C:C | C:C | C:C |
| 69 | wild-type | C:T | C:T | C:T | C:T | C:T | C:T |
| 70 | wild-type | C:T | C:T | C:T | C:T | C:T | C:T |
| 71 | wild-type | C:T | C:T | C:T | C:T | C:T | C:T |
| 72 | wild-type | C:T | C:T | C:T | C:T | C:T | C:T |
| 73 | wild-type | C:T | C:T | C:T | C:T | C:T | C:T |
| 74 | wild-type | C:T | C:T | C:T | C:T | C:T | C:T |
| 75 | wild-type | C:T | C:T | C:T | C:T | C:T | C:T |
| 76 | wild-type | C:C | C:C | C:T | C:T | C:T | C:T |
| 77 | wild-type | C:C | C:C | C:C | C:C | C:C | C:C |
| 78 | wild-type | C:T | C:T | C:T | C:T | C:T | C:T |
| 79 | wild-type | C:T | C:T | C:C | C:C | C:C | C:C |
| 80 | wild-type | C:C | C:C | C:C | C:C | C:C | C:C |
| 81 | wild-type | C:T | C:T | C:T | C:T | C:T | C:T |
| 82 | wild-type | C:T | C:T | C:T | C:T | C:T | C:T |
| 83 | wild-type | C:T | C:T | C:T | C:T | C:T | C:C |
| 84 | wild-type | C:T | C:T | C:T | C:T | C:T | C:T |
| 85 | wild-type | C:C | C:C | C:C | C:C | C:C | C:C |
| 86 | wild-type | C:T | C:T | C:T | C:T | C:T | C:T |
| 87 | wild-type | C:T | C:T | C:T | C:T | C:T | C:T |
| 88 | wild-type | C:T | C:T | C:T | C:T | C:T | C:T |
| 89 | wild-type | C:C | C:C | C:C | C:C | C:C | C:C |
| 90 | wild-type | C:C | C:C | C:T | C:T | C:T | C:T |
| 91 | wild-type | C:T | C:T | C:T | C:T | C:T | C:T |
| 92 | wild-type | C:T | C:T | C:T | C:C | C:T | C:T |
| 93 | wild-type | C:T | C:T | C:T | C:T | C:T | C:T |

**Table S6 List of primer sequence of KASPs**

| ID | Primer AlleleFAM | Primer AlleleHEX | Primer Common |
| --- | --- | --- | --- |
| *BraA10g000480.3C* | GAAGGTGACCAAGTTCATGCTTGAGTAACGAGACAAGAAAGATCAGTAC | GAAGGTCGGAGTCAACGGATTTGAGTAACGAGACAAGAAAGATCAGTAT | CGTTAACAGACAACACCGTTCAG |
| *BraA10g004420.3C* | GAAGGTGACCAAGTTCATGCTATTTCCCTCCTACTGAGGTGTTTC | GAAGGTCGGAGTCAACGGATTATTTCCCTCCTACTGAGGTGTTTT | TTACTGGATGGTTTTTGGGACAC |
| *BraA10g007580.3C* | GAAGGTGACCAAGTTCATGCTTCATCTGAATCATCATCTCTAGCTTC | GAAGGTCGGAGTCAACGGATTTCATCTGAATCATCATCTCTAGCTTT | CATACTGCAAGAAAAACCAGAGGA |
| *BraA10g008030.3C* | GAAGGTGACCAAGTTCATGCTATTTTGTATTCTGACTAGCTCAACAATAAG | GAAGGTCGGAGTCAACGGATTATTTTGTATTCTGACTAGCTCAACAATAAA | TGAAAAGGTAAACATTGCTTTTTGTAA |
| *BraA10g009700.3C* | GGGAGGATTCGAGGTTGTGG | GGGGAGGATTCGAGGTTGTGA | AGCTGACTGATGGCGATCCCTC |
| *BraA10g010270.3C* | GATAAAATGCTGCTTGAGCTTGAACG | AGATAAAATGCTGCTTGAGCTTGAACA | GCTGAGCTCTGCATCCATTAGCG |

**Table S7 The primers used for cloning, vector construction and qRT-PCR**

| Primer name | Primer Sequences (5′–3′) | Experiment |
| --- | --- | --- |
| *BraA10g000480.3C*-P1-F | ACTCCATGCTTATTCTGCGA | cloning promoter sequence |
| *BraA10g000480.3C*-P1-R  *BraA10g000480.3C*-P2-F  *BraA10g000480.3C*-P2-R | CACCGTTCCGTTATTACTCC  GCGTGGAGTAATAACGGAAC  ACGAGAGGAGACGGCTGT |  |
| *BraA10g000480.3C*-1-F | CACGACACCGACCGTAGAC | cloning DNA sequence |
| *BraA10g000480.3C*-1-R | GATTCAAGAAGCAGGAAAACAC |  |
| *BraA10g000480.3C*-2-F | TTGTTGCATGACTTCAAGTGG |  |
| *BraA10g000480.3C*-2-R | GAATTTTTTTGTACGTTAGGGG |  |
| *BraA10g000480.3C*-3-F | TTGAGGACCTTCAGCACCA |  |
| *BraA10g000480.3C*-3-R | TTTAGTAGATTCGGACCAAATATG |  |
| *BraA10g000480.3C*-F | ATGAGCAAGATCCGTTCGT | cloning cDNA sequence |
| *BraA10g000480.3C*-R | TCAATCGATCCAACGTGTG |  |
| *BraA10g000480.3C*-F1  *BraA10g000480.3C*-R1 | TGTCCTCTACTTTGATGTCCCTGA  TGAGTATCTGAACGGTGTTGTCTGT | qRT-PCR |
| *BrActin-F* | ATCTACGAGGGTTATGCT |  |
| *BrActin-R* | CCACTGAGGACGATGTTT |  |
| ANp-F | cagtTTTCGCAGCATCTAACGAGCTCTTCGcaactttctatgatgatttcaacg | Vector construction |
| ANp-R | cgatTTTCGCAGCATCTAACGAGCTCTTCGcttctcgctactcccgatga |  |
| pAN::BrAN:GFP-F  pAN::BrAN:GFP-R  pAN::Bran:GFP-F  pAN::Bran:GFP-R | cagtTTTCGCAGCATCTAACGAGCTCTTCGgaagatgagcaagatccgttcgtc  cagtTTTCGCAGCATCTAACGAGCTCTTCGtacatcaatcgatccaacgtgtga  cagtTTTCGCAGCATCTAACGAGCTCTTCGgaagatgagcaagatccgttcgtc  cagtTTTCGCAGCATCTAACGAGCTCTTCGtacatcaatcgatccaacgtgtga |  |
